# Novel LysM motifs for antigen display on lactobacilli for mucosal immunization

**DOI:** 10.1038/s41598-021-01087-8

**Published:** 2021-11-04

**Authors:** Fernanda Raya-Tonetti, Melisa Müller, Jacinto Sacur, Haruki Kitazawa, Julio Villena, Maria Guadalupe Vizoso-Pinto

**Affiliations:** 1 Infection Biology Laboratory, Instituto Superior de Investigaciones Biológicas (INSIBIO), CONICET-UNT, 4000 Tucumán, Argentina; 2 Laboratorio de Ciencias Básicas & Or. Genética, Facultad de Medicina, Universidad Nacional de Tucumán, 4000 Tucumán, Argentina; 3 Laboratory of Immunobiotechnology, Reference Centre for Lactobacilli (CERELA-CONICET), 4000 Tucumán, Argentina; 4 Food and Feed Immunology Group, Laboratory of Animal Food Function, Graduate School of Agricultural Science, Tohoku University, Sendai, 980-8572 Japan; 5 Livestock Immunology Unit, International Education and Research Center for Food and Agricultural Immunology (CFAI), Graduate School of Agricultural Science, Tohoku University, Sendai, 980-8572 Japan

**Keywords:** Protein delivery, Biotechnology, Immunology, Mucosal immunology, Vaccines, Vaccines, Microbiology, Bacterial techniques and applications

## Abstract

We characterized two LysM domains of *Limosilactobacillus fermentum*, belonging to proteins Acglu (GenBank: KPH22907.1) and Pgb (GenBank: KPH22047.1) and bacterium like particles (BLP) derived from the immunomodulatory strain *Lacticaseibacillus rhamnosus* IBL027 (BLPs027) as an antigen display platform. The fluorescence protein Venus fused to the novel LysM domains could bind to the peptidoglycan shell of lactobacilli and resisted harsh conditions such as high NaCl and urea concentrations. Acglu with five LysM domains was a better anchor than Pgb baring only one domain. Six-week-old BALB/c mice were nasally immunized with the complex Venus-Acglu-BLPs027 at days 0, 14 and 28. The levels of specific serum IgG, IgG1 and IgG2a and the levels of total immunoglobulins (IgT) and IgA in broncho-alveolar lavage (BAL) were evaluated ten days after the last boosting. Venus-Acglu-BLPs027, nasally administered, significantly increased specific BAL IgT and IgA, and serum IgG levels. In addition, spleen cells of mice immunized with Venus-Acglu-BLPs027 secreted TNF-α, IFN-γ and IL-4 when stimulated ex vivo in a dose-dependent manner. We constructed a Gateway compatible destination vector to easily fuse the selected LysM domain to proteins of interest for antigen display to develop mucosal subunit vaccines.

## Introduction

Novel vaccines against pathogens targeting the respiratory and gastrointestinal tracts are mandatory, particularly for high-risk populations such as children, the elderly and immunocompromised hosts since mortality rates from preventable infections are high in developing countries^1,2^. Intramuscular or subcutaneous vaccines induce a strong systemic immune response with high production of specific IgG antibodies but poor immune responses at mucosal sites. Therefore, they have a limited effect on the replication of pathogens at the site of entry^3^. As most infectious diseases start at the mucosal level, there is a need for oral or intranasal vaccines that offer protection at these sites. In addition, the elevated cost of vaccines restricts their implementation in massive health programs^4^. Mucosal vaccines are of easy administration (no specialized personnel needed) and painless, which contributes to the high adherence of patients. Therefore, they are more appropriate for mass vaccination in developing countries or during pandemics^5^.

The administration of antigens through the mucosal routes is challenging because it may generate tolerance rather than effector immune responses. Immunotolerance is the natural mucosal immune response to soluble antigens that has evolved to prevent harmful inflammatory responses^6^.

Lactic acid bacteria (LAB) with immunomodulatory properties, referred to as immunobiotics, efficiently modulate mucosal immune responses^7^. Since the 1990s, the use of LAB as a mucosal vaccine platform has been explored since they have both carrier and adjuvant properties^8^. In this sense, Dr. Klaenhammer's group pioneered the use of *L. acidophilus* and other LAB as vehicles for living bacteria to express and deliver antigens to the intestinal tract or to regulate the intestinal immune response. In this approach, food grade lactobacilli serve to first express, encapsulate and protect antigens during transit through the stomach and second to administer and deliver the vaccine in the gastrointestinal tract (reviewed in ^9^) However, there are regulatory hurdles associated with the use of vaccines that employ genetically modified organisms as carriers. We reported that *Lacticaseibacillus rhamnosus* IBL027 (Basonym: *Lactobacillus rhamnosus*) has both intrinsic antiviral immunomodulatory and mucosal adjuvant properties^10^. Further, the viability of LAB is not necessary to achieve the immunomodulatory effect^11^. In fact, we demonstrated that bacterium-like particles (BLPs) derived from the IBL027 strain (BLPs027) enhance local and systemic, humoral and cellular immune responses when co-administered with a mucosal viral vaccine^11^. BLPs are obtained by treating LAB with hot acid, resulting in bacterial death, loss of DNA and cytoplasmic proteins, and exposure of the cell wall peptidoglycan^11^, which can be used to deliver antigens^12^. In this sense, there are some studies where particles derived from *Lactococcus lactis* (known as GEM, gram-positive enhancer matrix) were used as mucosal delivery vehicles for heterologous antigens of bacterial, viral or parasitic nature^13^. These vaccines induce robust and long-lasting adaptive immune responses thanks to an optimal activation of innate immune responses through the Toll-like receptor 2 (TLR2)^14^.

Our goal was to develop a platform for mucosal vaccines exploiting the well described immunomodulatory potential of *L. rhamnosus* in combination with an antigen display system to serve not only as adjuvant but also as carrier without being a genetically modified organism (GMO).

The LysM motif is a cell wall binding domain widely distributed in nature. It is a ubiquitous 42–65 amino acids long motif found across prokaryotes and eukaryotes in more than 4,000 proteins. One to 12 LysM motifs specifically bind to N-acetylglucosamine residues of peptidoglycan non-covalently^15^ but there is some controversy on whether the number of LysM motifs in proteins affects the efficiency of binding to the BLPs^16^. Over 40 different antigens of bacterial, viral, or parasitic nature have been successfully overexpressed using the C-terminal peptidoglycan-binding domain of AcmA, an autolysin from *Lactococcus lactis*^17^.

In this study we cloned, expressed, and characterized two yet undescribed LysM domains. Then, the fusion protein constituted by Venus (a model antigen) and the newly characterized LysM domain were tested for their ability to bind to BLPs from *L. rhamnosus* IBL027. Finally, the mucosal and systemic immune responses elicited by the experimental vaccine, i.e., BLPs exposing the antigen bound to their peptidoglycan, were studied in intranasally immunized mice.

## Results

### Expression and purification of two novel LysM proteins

The LysM domains of two proteins, Acglu (GenBank: KPH22907.1) and Pgb (GenBank: KPH22047.1), selected from Pfam database were subcloned in the destination vector pETG-N-RGS-His-Venus-[rfB] using the Gateway Technology according to the manufacturer’s instructions and as depicted in Fig. S1. We aligned AcmA, MurO and Sep proteins, and the LysM motifs of the undescribed proteins Pgb and Acglu using the tool T-COFFEE. The alignment revealed a highly conserved sequence among the different LysM domains (Fig. S6A).

The recombinant proteins His-Venus-Acglu (64 KDa) and His-Venus Pgb (35 KDa) were expressed in *E. coli* Rosetta after induction with 2 mM IPTG. In both cases, greater amount of protein was found in the inclusion bodies. The proteins were purified using NiNTA chromatography and checked by SDS-PAGE and western blotting using a mouse monoclonal anti-RGS-His antibody^18^ (Fig. S2).

### LysM domains efficiently bind to BLPs027

**Figure 1:**
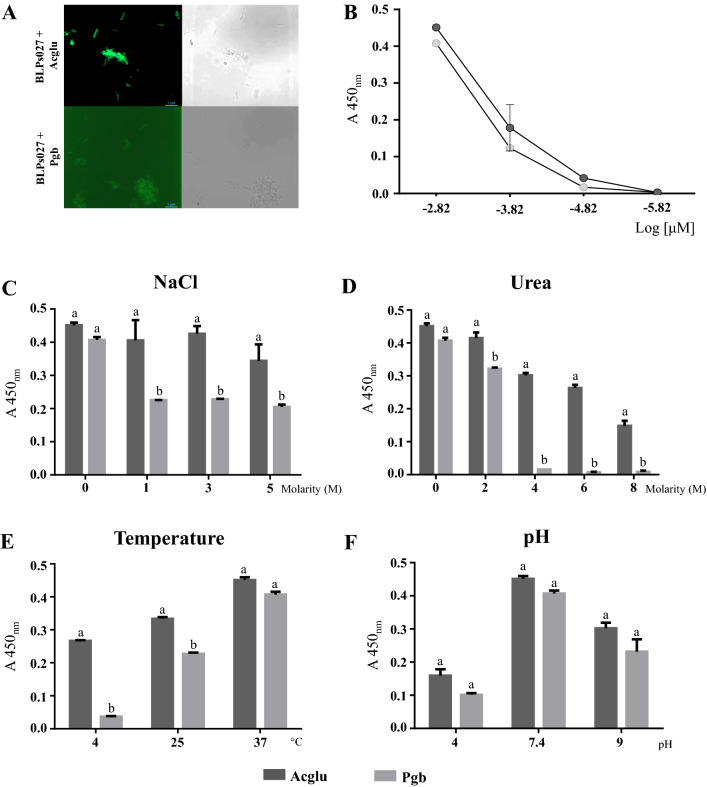
Binding of Venus-LysM proteins to bacterium-like particles (BLPs). **(A)** Fluorescence microscopy analysis of Venus-Acglu and Venus-Pgb proteins obtained from the inclusion bodies bound to BLPs from *Lacticaseibacillus rhamnosus* IBL027 (BLPs027)**. (B)** Detection of binding affinity of different concentrations of Venus-Acglu and Venus-Pgb to BLPs027 by ELISA. **(C)** Influence of NaCl molarity (1 M, 3 M and 5 M), **(D)** urea molarity (2 M, 4 M, 6 M and 8 M), **(E)** temperatures (4, 25 and 37 °C), and **(F)** pH (4, 7.4 and 9) on the binding affinity of Venus-Acglu and Venus-Pgb proteins to the BLPs027. Different letters above bars indicate significant differences between groups in the same condition. P < 0.05 was considered significant.

Venus green fluorescence was observed on the surface of BLPs027 treated with both proteins separately, being the intensity of Acglu-Tagged Venus fluorescence higher than Pgb-tagged Venus suggesting a higher amount of bound protein in the former (Fig. 1A). This experiment was performed with the cell-free crude extract in which the amount of protein may have been different. Thus, we adapted an ELISA-based technique (Fig. S3) in order to compare the binding properties of purified Acglu and Pgb to BLPs027 at equimolar concentrations.

As expected, the ELISA signal was proportional to the concentration of the LysM fusion proteins added to the BLPs. Due to solubility issues, we could not test higher concentrations and therefore the binding curve did not reach a plateau. These results showed that the binding capacity of Acglu and Pgb was similar (Fig. 1B) in contrast to the binding stability of LysM fusion proteins to BLPs027 that vary under different conditions. After protein binding, LysM-fusion-protein-BLP complexes were washed three times using buffers with either different NaCl molarity (1, 3, and 5 M), urea molarity (2, 4, 6, and 8 M) or pH (4, 7.4, and 9). When NaCl was included in the washing buffer, Pgb was partially washed out with 1 M NaCl, whereas Acglu remained attached to BLPs and was partially washed at a fivefold higher concentration of NaCl (Fig. 1C). Similarly, 2 M urea was able to detach Pgb from BLPs (Fig. 1D), and with 4 M urea practically all this protein was washed out from the particles. In the case of Acglu, much higher concentrations of urea were needed to detach this protein from the particles. Surprisingly, even at concentrations as high as 8 M, significant amounts of Venus-Acglu remained bound to the BLPs after the washing steps (Fig. 1D).

In another set of experiments, protein binding was conducted at different temperatures (4, 25 and 37 °C) with TBS-T as washing buffer. No significant differences were observed in the ability of both proteins to bind to the particles at 37 °C. However, Acglu showed a greater ability to bind to particles at 4 and 25 °C compared to Pgb. At 4 °C Pgb was almost undetectable on the surface of BLPs whereas a considerable amount of Acglu still bound to particles at this temperature (Fig. 1E). No differences in binding were observed between the proteins at different pH (Fig. 1F).

### Construction of an Acglu-tagged expression vector

Considering previous results, the five-repeats LysM domain of Acglu was selected to construct a bacterial expression vector compatible with Gateway (Fig. S4), to produce fusion proteins. The final expression vector pENHAC-[rfB] is a derivative of pETG-N-RGS-His-[rfB], in which the Acglu sequence was added upstream of the *ccdB gene* (Fig. S6B).

### Development of a LysM-BLPs027-based experimental vaccine

**Figure 2:**
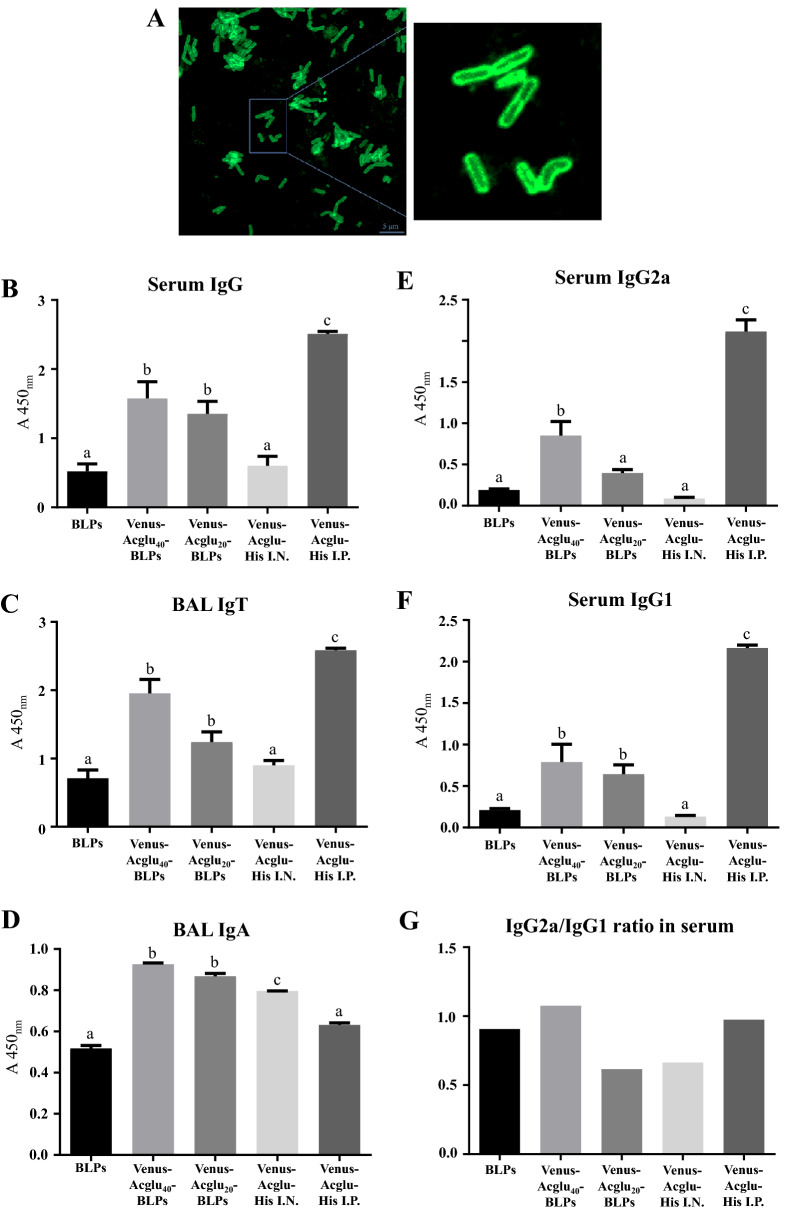
Immunogenicity of Venus-Acglu-BLPs027 experimental vaccine. **(A)** Fluorescence microscopy analysis of Venus-Acglu obtained under denaturing conditions bound to BLPs027. **(B–D)** Ten days after the boosting, serum, and BAL samples were obtained for the determination of IgG, total immunoglobulin (IgT), and IgA specific antibodies **(E–G).** Ten days after the 2nd boosting, serum samples were obtained for the determination of IgG1 and IgG2a specific antibodies. The IgG2a/IgG1 ratio was also calculated. Each experimental group consisted of five mice per group. Different letters above bars indicate significant differences between groups. P < 0.05 was considered significant.

We next aimed to develop an experimental vaccine by using the LysM domains of Acglu, the BLPs027 and a model antigen. We selected Venus considering that it is immunogenic and that offers experimental advantages due to its fluorescence. The cell-free extract containing Venus-Acglu was mixed with BLPs027, and after washing, the particles were subjected to SDS-PAGE analysis and compared to BSA standards. Densitometry analysis of the bands by ImageJ indicated that 10^8^ BLPs027 could bind a maximum of 40 μg of Venus-Acglu. Venus-Acglu displayed on BLPs027 was confirmed by fluorescence microscopy (Fig. 2A).

### Immunogenicity of the LysM-BLPs027-based experimental vaccine

To investigate whether the experimental Venus-Acglu-BLP027 vaccine was immunogenic, experiments were performed in immunocompetent adult mice. For this purpose, two different formulations were prepared using 20 and 40 µg of Venus-Acglu and BLPs027: Venus-Acglu_20_-BLPs027 and Venus-Acglu_40_-BLPs027, respectively. Mice were immunized as described in 2.8.

We found that His-tagged Venus-Acglu is immunogenic when administered with complete Freund's adjuvant by the intraperitoneal route, since Venus-specific serum IgG antibodies were detected (Fig. 2B). Moreover, Venus-specific BAL IgT and IgA antibodies were detected in BAL samples (Fig. 2C,D). As expected, the nasal immunization with Venus-Acglu-His was less efficient than the intraperitoneal immunization with Freund’s adjuvant in generating specific systemic humoral immune response even after the second boosting (Fig. 2B). Mice in the Venus-Acglu_20_-BLPs027 and Venus-Acglu_40_-BLPs027 groups had higher levels of specific serum and respiratory antibodies than the mice which received Venus-Acglu-His nasally (Fig. 2B,D). The levels of IgT were higher than those of IgA in all experimental groups (Fig. 2C,D). Interestingly, the experimental intranasal vaccine was able to increase the IgA levels in a dose-dependent manner when compared to the control group that received the vaccine with Freund's adjuvant intraperitoneally suggesting that the Venus-Acglu-BLPs027 complex not only stimulates a Venus-specific mucosal immunity but is more efficient than the intraperitoneal vaccine to achieve this purpose (Fig. 2D).

Both Venus-Acglu_20_-BLPs027 and Venus-Acglu_40_-BLPs027 induced higher levels of serum specific IgG1 antibodies when compared to mice immunized with Venus-Acglu-His by the nasal route (Fig. 2F); in contrast, only the Venus-Acglu_40_-BLPs027 immunization generated higher serum IgG2a levels than control mice (Fig. 2E). All the groups immunized by the nasal route showed levels of IgG1 and IgG2a that were significantly lower than the animals that received Venus-Acglu-His by intraperitoneal injection (Fig. 2E,F). Interestingly, the IgG2a/IgG1 ratios in mice nasally immunized with Venus-Acglu_20_-BLPs027 and Venus-Acglu-His were 0.6 and 0.5, respectively, while immunization with Venus-Acglu_40_-BLPs027 induced an IgG2a/IgG1 ratio of 1.1, suggesting an improved Th1 response in the latter group (Fig. 2G).

**Figure 3:**
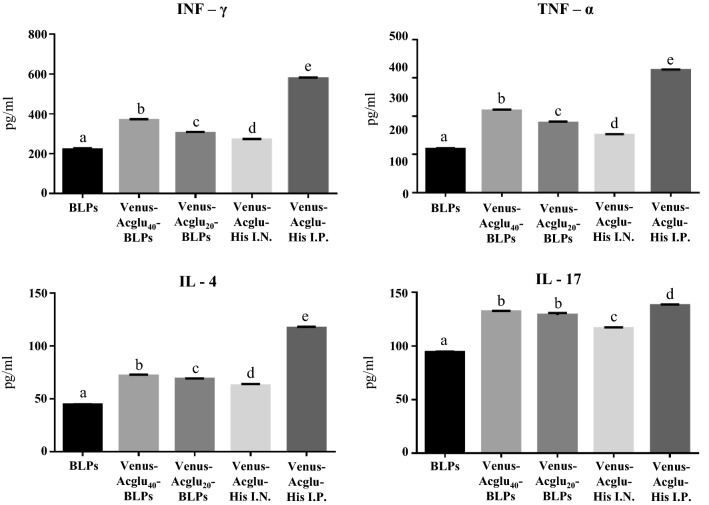
Immunogenicity of Venus-Acglu-BLPs027 experimental vaccine. Spleen samples were taken ten days after the boosting and immune cells were isolated. Cultured spleen immune cells were challenged ex vivo with His-Venus antigen and the concentrations of tumor necrosis factor (TNF)-α, interferon (IFN)-γ, interleukin (IL)-4 and IL-17 were determined in supernatants after 24 h of stimulation. Each experimental group consisted of five mice per group. Different letters above bars indicate significant differences among groups. p < 0.05 was considered significant.

To evaluate the cellular immune response induced by the Venus-Acglu-BLPs027 experimental vaccines, we then assessed the ability of immune cells isolated from the spleens of vaccinated mice to produce cytokines in response to the ex vivo stimulation with the His-tagged Venus protein. The levels of TNF-α, IFN-γ, IL-4 and IL-17 were evaluated in the supernatants of antigen-stimulated splenocytes (Fig. 3). Immune cells isolated from the spleens of mice immunized with His-tagged Venus-Acglu with complete Freund's adjuvant produced the four cytokines tested (TNF-α, IFN-γ, IL-4 and IL-17) in response to the antigen challenge. In addition, we found that the production of the four cytokines was significantly lower in mice nasally vaccinated with Venus-Acglu-His than the animals immunized intraperitoneally, even after boosting (Fig. 3). Levels of TNF-α, IFN-γ, IL-4 and IL-17 were significantly higher in cells from Venus-Acglu_20_-BLPs027 or Venus-Acglu_40_-BLPs027 immunized mice than the observed in the nasal His-tagged Venus-Acglu group. We observed a dose-depending effect: spleen cells derived from mice immunized with 40 µg produced higher levels of TNF-α, IFN-γ and IL-4 in response to the antigen stimulation than the spleen cells from mice immunized with 20 µg (Fig. 3).

## Discussion

Mucosal vaccination is highly desired for improving protection against infectious diseases since most pathogens enter and initiate their replication at the mucosal surfaces^19^. In this regard, the intranasal delivery of vaccines allows for non-invasive, practical, simple, and inexpensive administration of antigens. Additionally, the large surface area and the rich capillary plexuses of the nasal mucosal tissue contribute to the quick absorption of antigens influencing systemic immunity as well^20^.

The immunomodulatory effects of LAB were combined with antigen delivery using recombinant technologies to develop experimental mucosal vaccines as an alternative to viable genetically modified LAB^21,22^. In this regard, *L. lactis*-derived BLPs, (previously described as GEM), efficiently deliver pathogenic antigens to mucosal tissues. Most of the GEM-based vaccines formulated so far use a peptidoglycan-binding domain derived from the C-terminal part of the lactococcal cell-wall hydrolase AcmA^15^.

We hypothesized that this BLPs-based delivery/adjuvant system for heterologous antigens can be improved by: (a) selecting LAB that give rise to BLPs with better immunoadjuvant properties, (b) increasing the capacity of antigen binding to the surface of BLPs and, (c) generating more stable antigen-BLPs joints that efficiently resist harsh conditions found at mucosal surfaces. Recently, we have demonstrated that BLPs originated from different immunomodulatory lactobacilli significantly differed in their adjuvant capacities^11^. BLPs from the immunomodulatory strain *L. rhamnosus* IBL027 had strong adjuvant properties when orally administered with a commercial rotavirus vaccine while the *L. plantarum* CRL1506 BLPs had minimal impact in the immune responses induced by the oral vaccination. Considering that AcmA contains three LysM domains^23,24^, we aimed to test two alternatives by varying the number of LysM domains added to the recombinant antigens, and using proteins derived from lactobacilli. Then, we selected the only LysM domain encoded in the Pgb protein and five LysM domains encoded in the Acglu protein of *L. fermentum* IBL038.

Buist et al. postulated that the number of LysM domains affect the binding capacity of proteins to peptidoglycan^16^. In agreement, it was reported that recombinant antigens carrying one LysM motif from AcmA have low binding affinity to peptidoglycan while two LysM motifs were sufficient to obtain a binding affinity similar to the native AcmA protein. In contrast, the peptidoglycan hydrolase AcmD from *L. lactis*, which is a homolog of the autolysin AcmA and contains three LysM sequences, exhibited poor binding to GEM particles compared to AcmA^15^. These results indicated that the binding affinity not only depends on the number of LysM motifs. In fact, it was shown that the extracellular protein Sep from *L. fermentum* BR11, which contains a single LysM domain, is a useful heterologous peptide fusion partner^25^. In our study, we observed that the addition of the five LysM motifs from Acglu to the recombinant protein did not confer a greater capacity of binding to BLPs when compared to the single LysM motif of Pgb at equal micromolar concentrations. Nevertheless, we found that the higher number of LysM motifs present in Acglu conferred a greater resistance to adverse conditions such as high concentrations of urea and NaCl. Interestingly, no differences between the amount of Acglu and Pgb attached to BLPs were observed when the pH values were modified. Near-field scanning optical microscopy and atomic force microscopy studies demonstrated a strong and highly stable binding of AcmA to BLPs at physiological pH^26^. It was also reported that there was an abrogation of this specific binding when the pH was reduced to 4.4, an acidic environment that can be found in the phagolysosomes of antigen presenting cells. Thus, our results indicate that the incorporation of LysM motifs from Acglu or Pgb to recombinant antigens could have a practical advantage over the LysM motifs of AcmA by providing greater stability during the antigen presentation process.

We also evaluated whether Venus-Acglu-BLPs027 could induce specific immune responses when nasally administered to adult immunocompetent mice. Nasal immunizations with two different doses of the complex Venus-Acglu-BLPs027 were compared with the recombinant protein Venus-Acglu-His alone. Interestingly, although there was a tendency of the Venus-Acglu_40_-BLPs027 immunization to induce higher levels of respiratory IgT and serum IgG antibodies when compared to the Venus-Acglu_20_-BLPs027 treatment, there were no statistically significant differences between the groups. Some studies indicated that the concentration of antigens attached to the GEM particles did not modify the stimulation of the humoral immune response. Experiments in mice nasally immunized with GEM particles carrying three different doses of the pneumococcal antigen PspA3 did not generate differences in the titers of serum IgG or BAL IgA antibodies but the highest dose was superior than the others in terms of improving survival of *S. pneumonaie* infected mice^27^. In previous experiments, we observed that administering different doses of BLP to adjuvate a rotavirus live vaccine resulted in different levels of stimulation ^11^. Similarly, in this study, there were differences with higher antigen doses: Venus-Acglu_40_-BLPs027 induced significantly higher levels of serum specific IgG1 antibodies resulting in a higher IgG2a/IgG1 ratio when compared to Venus-Acglu_20_-BLPs027. Thus, a higher dose of the Venus-Acglu antigen may stimulate more efficiently the cellular immune response. Other studies reporting LAB-based vaccines have evaluated the effect of mucosal immunizations by determining antigen-specific antibody responses ^21^, while cellular immunity was not investigated. The stimulation of cellular immunity is recognized as a key factor for the prevention of viral and bacterial infectious diseases. Indeed, the WHO guidelines recommend measuring cellular immunity as a part of their endpoints in clinical trials^28^.

To evaluate cellular immune responses to our experimental vaccine, we studied the cytokine response of splenocytes isolated from vaccinated mice followed by an ex vivo challenge with Venus and determined that the higher dose of Venus-Acglu_40_-BLPs027 was better to stimulate cellular immunity. Further, the splenocytes of these mice produced higher levels of TNF-α, IFN-γ, and IL-4 in response to the specific antigen stimulation than the Venus-Acglu_20_-BLPs027 group. The uptake of microorganisms by antigen presenting cells results in the early production of TNF-α. A strong TNF-α response by macrophages and DCs have been associated to improved Th1-mediated immunity^23,29^. BLPs alone or displaying antigens stimulated the production of TNF-α in DCs of both adult and neonatal mice^30^. Furthermore, intranasal immunizations with BLPs mixed with a split influenza vaccine promoted the development of specific IFN-γ T cells in local lymphoid nodes and spleen ^31^ and induced a shift from a balanced Th1/Th2 to a predominant Th1-type response^32^. The ability of vaccines formulated with BLPs to induce high levels of antigen-specific IFN-γ in mucosal tissues is a remarkable property considering that such responses are difficult to be induced early in life^30^.

Two important factors influence the immune responses elicited by mucosal vaccines: the choice of a proper adjuvant and the route of administration. It is not surprising that Venus-Acglu-His intraperitoneally administered with complete Freund's adjuvant elicited higher systemic immune responses than antigens delivered nasally, because of the high toxicity of this adjuvant whose use in humans is forbidden. Several studies reported a lower efficiency of nasally administered GEM-based vaccines to induce systemic immunity when compared to subcutaneous, intramuscular or intraperitoneal delivery of antigens^33,34^. When *L. lactis*-BLPs-based vaccines for influenza^35^, respiratory syncytial virus^36^ and *S. pneumoniae*^27^, were administered intranasally, increases in IgA levels were observed compared to the groups of animals that received them intraperitoneally. This increase was correlated with a superior protection against the pathogens since, in all cases, their titers were lowered in the lungs. These results suggest a fundamental role of local immunity in the protective capacity of intranasal vaccines based on this platform. Consistent with these results, our experimental vaccine induced mucosal IgA in mice in a dose-dependent manner. This indicates that an antigen bound to BLPs027 and administered intranasally not only induces systemic humoral immune responses but also improves mucosal immunity compared to the vaccine administered intraperitoneally. Nevertheless, further detailed studies evaluating the effect of nasally administered Antigen-Acglu-BLPs027 complexes using pathogenic antigens of viral or bacterial origin are a mandatory next step to position our Acglu-BLPs027 system as a potential tool in the generation of mucosal vaccines.

Vaccines are the most cost-effective and suitable means of controlling infectious diseases. Thus, developing cost-effective vaccine strategies that could provide a stronger immune response with reduced vaccination schedules and maximum coverage is of critical importance. From an application point of view, the LysM domain can be taken out of its natural context and fused with other proteins. Therefore, it is possible to bind a protein of interest to the peptidoglycan-rich wall of Gram-positive bacteria. We propose the presentation of recombinant proteins on the surface of IBLP027 to be used as subunit mucosal vaccines, both for reasons related to the stability and accessibility of the presented protein and the low cost of this platform.

## Materials and methods

### Bacterial strains and growth conditions

*L. fermentum* IBL038 and *L. rhamnosus* IBL027 deposited in the Faculty of Biochemistry, Chemistry and Pharmacy of the National University of Tucumán (Tucumán, Argentina), were grown for 12 h at 37 °C in Man-Rogosa-Sharpe (MRS) broth (final log phase).

*E. coli* strains (TOP10, DH5α, DB3.1 and Rosetta) were cultured in Luria Bertani broth or on agar plates at 37 °C supplemented with 100 µg/ml ampicillin, 50 µg/ml gentamicin or 17 µg/ml chloramphenicol for plasmid selection.

### PCR amplification of LysM domains

We selected two proteins of *Limosilactobacillus fermentum* (Basonym *Lactobacillus fermentum*) from the Pfam database not described before in the literature: Peptidoglycan-binding protein (Pgb) (GenBank: KPH22047.1) with one LysM domain and Mannosyl-glycoprotein endo-beta-*N*-acetylglucosamidase (Aclgu) (GenBank: KPH22907.1) with five LysM domains. We amplified the LysM sequences using a nested PCR as described before^37^ to build the Entry clones (Gateway) following the manufacturer’s instruction depicted in Fig. S1. The primers used include a fragment of the attB1 and attB2 sites for the nested PCR to build the Entry clones (Gateway) (Table 1). *L. fermentum* IBL038 genomic DNA was used as a template to run the PCR reaction with the *Pfu* polymerase using the corresponding annealing temperatures (Table 1). A second nested PCR was done to complete the attB sites as described before ^37^. The specific PCR products were isolated with the commercial kit High pure PCR product purification (Roche).

**Table 1:** Primers used to build the Entry library (Gateway) and their corresponding annealing temperatures.

Lf-Acglu-Fw	AAAAAGCAGGCTCCGCCATGGTCCAATCCGGCGACAC	56 °C
Lf-Acglu-Rv	AGAAAGCTGGGTCAAGCGATAACTGTTGACC	56 °C
Lf-Pgb-Fw	AAAAAGCAGGCTCCGCCATGATTTACACCGTTAAGAGTGG	54 °C
Lf-Pgb-Rv	AGAAAGCTGGGTCGATCACTAACTTTTGCCC	54 °C

The LysM motifs of the *N*-acetylmuramidase (AcmA from *L. lactis*), of the gamma-D-glutamyl-meso-diaminopimelate peptidase (MurO from *L. plantarum*), of the extracellular surface protein (Sep from *L. fermentum*), of Pgb (*L. fermentum*) and of Acglu (*L. fermentum*) were aligned with the T-COFFEE program, version 11.00.d625267.

### Gateway recombinant cloning of LysM domains and expression of the chimeric proteins

The Gateway cloning technology (Life Technologies) was used to introduce the sequences of interest, Pgb and Acglu, into the bacterial expression vector pETG-N-His-Venus-[rfb] according to the manufacturer's instructions (Fig. S1). Briefly, a nested PCR was performed to amplify the desired gene as described in 4.2. Then, the reaction mix was prepared with 1 µl BP-clonase (Life technologies) containing 3 µl of the purified PCR product, and 1 µl of pDONR207, incubated at room temperature overnight and subsequently transformed by heat shock in chemically competent *E. coli* Top10. Plasmid DNA from individual colonies grown on LB plates supplemented with 50 μg/ml gentamicin (Life Technologies) was isolated using the High Purity Plasmid Isolation Kit (Roche) and the integrity of the pENTRY207-LysM resulting vectors was verified by enzymatic restriction with *Ban*II (New England Biolabs) and sequenced at the CERELA-CONICET (Tucumán, Argentina) sequencing service. After this, LR recombination reactions, using the LR-clonase II enzyme mixture (Life Technologies), were performed according to the manufacturer's instructions (Fig. S1). Briefly, the pENTRY207-LysM vectors were recombinantly cloned into the pETG-N-RGS-His-Venus- [rfB] vector. LR clonase reactions containing 1 µl of pENTRY207-LysM, 1 µl of pETG-N-RGS-His-Venus-[rfB], 2 µl of molecular grade H_2_O, and 1 µl of the enzyme, were incubated at room temperature overnight and subsequently transformed into chemically competent *E. coli* Top10. Plasmid DNA from individual colonies grown on LB plates supplemented with 100 μg/ml ampicillin (Sigma-Aldrich, Germany) was isolated as described above and the integrity of the resulting pETG-N-RGS-His-Venus-LysM vectors was verified by enzymatic restriction analysis with *Hind*III and *Xba*I and gel electrophoresis (New England Biolabs, Germany).

Finally, the *E. coli* Rosetta strain was transformed with the expression vectors pETG-N-RGS-His-Venus-Acglu or pETG-N-RGS-His-Venus-Pgb by heat shock and the clones were selected on plates with LB medium supplemented with 100 μg/ml ampicillin and 17 μg/ml chloramphenicol. Individual colonies were selected, and the production of the recombinant protein was evaluated. The LB broth added with antibiotics was inoculated the transformed strain. After growing to A_560nm_ = 0.3 at 37 °C with shaking, the protein expression was induced with 2 mM IPTG, until an A_560nm_ = 0.7–0.8 was reached. The cultures were centrifuged at 4 °C to separate the cell pellet from the supernatant. Bacterial pellets were resuspended with ice cold lysis buffer (20 mM Tris–HCl; 0.5 M NaCl; 10% glycerol and 5 mM imidazole, pH 7.9) supplemented with 0.02 mg/ml DNAse, 0.1% Triton, 0.2 mM PMSF, 1 mM DTT and 1 mg/ml lysozyme and incubated on ice for 1 h 40 min until a clear lysate was obtained. The supernatant was separated by centrifugation at 4000×*g* for 30 min at 4 °C and it was kept as the soluble fraction called NC (native conditions) at − 20 °C. Then, the inclusion bodies were solubilized in a buffer containing 0.5 M NaCl, 5 mM imidazole, 20 mM Tris–HCl and 8 M urea at pH 7.9 (1 ml/g pellet) and incubated on ice 90 min with stirring. Finally, it was centrifuged for 30 min at 4000×*g* 4 °C, and the supernatant, called DC (denaturing conditions), with the insoluble proteins, was stored at − 20 °C. Expression and purity of recombinant proteins were analyzed by SDS-PAGE followed by staining with Coomassie Brilliant Blue and checked by Western blotting using a mouse monoclonal anti-RGS-His antibody (Qiagen). Both proteins were purified using NiNTA chromatography (Thermo Fisher Scientific) and stored at − 70 °C. Protein concentrations were determined with Bradford’s reagent (BioRad) following the manufacturer’s instructions.

### Preparation of BLPs, protein binding to BLPs and fluorescence microscopy

BLPs from *L. rhamnosus* IBL027 (BLPs027), an immunomodulatory strain, were prepared as described elsewhere^11^.

The purified recombinant Venus-Pgb or Venus-Acglu, or the crude extracts were incubated with BLPs (2.5 × 10^9^ BLP/ml) under gentle rotation for 1 h at room temperature. To remove unbound proteins, the particles were washed with sterile PBS and centrifugated at 13,000×*g* for 10 min three times. Finally, BLPs carrying Venus fused to LysM domains were resuspended in PBS and stored at − 70 °C.

The binding capacity of the recombinant fusion proteins to BLPs027 was evaluated by fluorescence microscopy. For that purpose, 10 μl of the BLP-LysM suspension was let air-dry on a microscope slide. After mounting with Vectashield (Vector, Burlingame, CA) the specimens were examined by fluorescence microscopy using a confocal microscope (LSM 800, Zeiss).

### ELISA and stability of the proteins bound to BLPs

An ELISA (Enzyme-Linked ImmunoSorbent Assay) was adapted from Petrovic et al.^38^, as shown in Fig. S3. Briefly, the BLPs were washed in coating buffer (100 mM NaHCO_3_, pH 9.6) and suspended in the same buffer at a concentration of 2.5 × 10^9^ particles/ml. Then, 100 µl of the BLP suspension was added to a 96-well polystyrene plate and incubated overnight at 37 °C. The wells were blocked with 1.5% gelatin (Sigma-Aldrich) in Tris buffer added with 1% Tween (TBST) for 1 h 30 min at 37 °C and subsequently washed with TBST as shown in Fig. S3. Then, 50 µl of the purified Venus-LysM fusion proteins (Acglu or Pgb) were added, with concentrations ranging between 1.5 × 10^–3^ and 1.5 × 10^–6^ µmol/ml, incubated for 2 h at 37 °C and subsequently they were washed three times with TBST. Finally, 100 µl of the diluted murine α-RGS-HIS antibody (1:2000, Sigma-Aldrich) was added, followed by a 2 h incubation at 37 °C and three washing steps with TBST. Peroxidase-conjugated anti-mouse antibody (α-Mo Pox 1:1000, Dako) was added and incubated for 1 h at 37 °C. After three TBST washing steps, the reaction was developed using TMB (Sigma-Aldrich) as a substrate and the A_450_ nm was measured on a microplate reader.

The stability of the proteins bound to LysM was determined under different conditions, including temperature (4, 25 and 37° C), NaCl molarity (1, 3, and 5 M), urea molarity (2, 4, 6, and 8 M) and pH (4, 7.4 and 9). For this purpose, different wash buffers were prepared using the mentioned conditions and after adding the solutions of the respective purified LysM proteins (1.5 × 10^–3^ μmol/ml), the wells were washed three times using the corresponding buffer. The final wash was done with TBST and the ELISA protocol was continued as previously described (Fig. S3).

### Construction of a customized pDEST vector

In order to label an antigen of interest with the Acglu domain, a personalized pDEST vector was constructed by traditional cloning following the procedure detailed in Fig. S4. The five LysM domains in Acglu were amplified from *L. fermentum* IBL038 genomic DNA using the following pair of primers: Acglu5-Fw: GGTAAGCTTATAGGAGGGCCACCATGAGAGGATCTCACCACCACCACGTCCAATCCGGCGACAC and Acglu5-Rv: GATATCACAAGCGATAACTGTTGACC. The purified product, 5'-*Hin*dIII-ATG-[RGS-His-tag-Acglu]-*Eco*RV-[ccdB/CmR (rfb)]-*Eco*RV-*Xba*I-3', was ligated into the pETG-N-RGS-His-[rfb] vector by using a T4 DNA ligase (New England Biolabs). Reactions were incubated overnight at 4 °C and subsequently transformed into chemically competent *E. coli* DB3.1 the integrity of the resulting pENHAC-[rfb] vector was verified by *Hind*III/*Xba*I (New England Biolabs) restriction analysis.

### Preparation of Acglu-Venus-BLP027 vaccine

Venus was cloned into the pENHAC-[rfB] vector using LR clonase (Gatewayrecombinatorial cloning) as described before. The recombinant fusion protein Acglu-Venus was induced in *E. coli* Rosetta with 2 mM IPTG. After sonication and centrifugation, the cell-free extract was mixed with BLPs027 as described in 4.4. The amount of bound protein was compared to BSA standards by SDS-PAGE.

### Immunization of mice

Six-week-old male Balb/c mice were obtained from a closed colony kept at CERELA-CONICET (Tucuman, Argentina). Five groups of five animals each were housed separately according to each treatment. All groups were fed a conventional balanced diet ad libitum.

Group 1, used as control group, only received 10^8^ particles/mouse resuspended in 20 µl of PBS. Groups 2 and 3 were immunized intranasally with Venus-Acglu-BLPs027 (10^8^ particles/mouse) using 20 or 40 μg of Venus-Acglu (Venus-Acglu_20_-BLPs027 and Venus-Acglu_40_-BLPs027 groups, respectively). Group 4 was nasally immunized with 40 μg of Venus-Acglu alone and group 5 was immunized intraperitoneally with 20 μg of Venus-Acglu with 100 µl complete Freund's adjuvant (1 mg/ml, Sigma-Aldrich). All groups of animals were immunized on day 0 and then received two booster vaccinations on days 14 and 28 (Fig. S5).

### Antibody detection in serum and broncho-alveolar lavage (BAL) and splenocytes cytokines production

Blood and BAL samples were taken 10 days after 2nd boosting as described previously ^39^.

Specific anti-Venus total immunoglobulins (IgT), IgA and IgG were determined by ELISA. Plates were coated with 3 μg recombinant His-Venus per well overnight at 4 °C and blocked with BSA. Appropriate dilutions of the samples (serum 1:20; BAL 1:2) were incubated for 1 h at 37 °C. Peroxidase conjugated anti-mouse IgT (Polyclonal rabbit anti-mouse immunoglobulins/HRP, DakoCytomation) IgG, or IgA antibodies (Sigma-Aldrich) (at a 1:500 dilution) were added and incubated for 1 h at 37 °C. The reaction was developed with TMB Substrate Reagent (Sigma-Aldrich) and measured at 450 nm in a microplate reader.

Spleens were collected, mechanically disaggregated and the cell suspensions were kept as previously described ^11^. Cells (4 × 10^6^ cells/well) were cultured in 24-well plates in the presence of 0.5 μg of His-Venus for 24 h. Tumor necrosis factor (TNF)-α, interferon (IFN)-γ, interleukin (IL)-4 and IL-17 concentrations in spleen supernatants were measured with commercially available ELISA kits following the manufacturer's recommendations (R&D Systems).

### Statistical analysis

Experiments were performed in triplicate and results were expressed as mean ± standard deviation (SD). After verification of the normal distribution of data, two-way ANOVA was used. Tukey's test (for pairwise comparisons of the means) was used to test for differences between the groups. Differences were considered significant at p < 0.05.

### Ethical statement

All methods were carried out in accordance with relevant guidelines and regulations. Animal experiments were performed in strict accordance with the ARRIVE guidelines. All experiments were approved by the Ethical Committee of Animal Care at CERELA-CONICET.

## Supplementary Information

Supplementary Figures.

## Data Availability

The datasets generated during and/or analysed during the current study are available from the corresponding author on reasonable request.
