# Supplementary material for: Novel LysM motifs for antigen display on lactobacilli for mucosal immunization
Source: Sci Rep. 2021 Nov 4;11:21691. doi: 10.1038/s41598-021-01087-8 (PMC8568972; doi:10.1038/s41598-021-01087-8)
Supplement: Supplementary file 1 — Supplementary Figures. [file 41598_2021_1087_MOESM1_ESM.pdf]

Novel LysM motifs for antigen display on lactobacilli for mucosal immunization

Fernanda Raya-Tonetti[1,2], Melisa Müller[1,2], Jacinto Sacur[1,2], Haruki Kitazawa[4,5*], Julio Villena[3,*,†] and Maria Guadalupe Vizoso-Pinto[1,2,*,†]

[1] Infection Biology Lab, Instituto Superior de Investigaciones Biológicas (INSIBIO), CONICET-UNT, 4000 Tucumán, Argentina.

[2] Laboratorio de Ciencias Básicas & Or. Genética, Facultad de Medicina, Universidad Nacional de Tucumán, 4000 Tucumán, Argentina

[3] Laboratory of Immunobiotechnology, Reference Centre for Lactobacilli (CERELA-CONICET), 4000 Tucumán, Argentina.

[4] Food and Feed Immunology Group, Laboratory of Animal Food Function, Graduate School of Agricultural Science, Tohoku University, Sendai 980-8572, Japan.

[5] Livestock Immunology Unit, International Education and Research Center for Food and Agricultural Immunology (CFAI), Graduate School of Agricultural Science, Tohoku University, Sendai 980-8572, Japan

[†] Equal contribution,

Corresponding authors:

Dr. Maria Guadalupe Vizoso Pinto
mgvizoso@fm.unt.edu.ar

Dr. Julio Villena
Jcvillena@cerela.org.ar

Dr. Haruki Kitazawa
haruki.kitazawa.c7@tohoku.ac.jp

Supplementary information

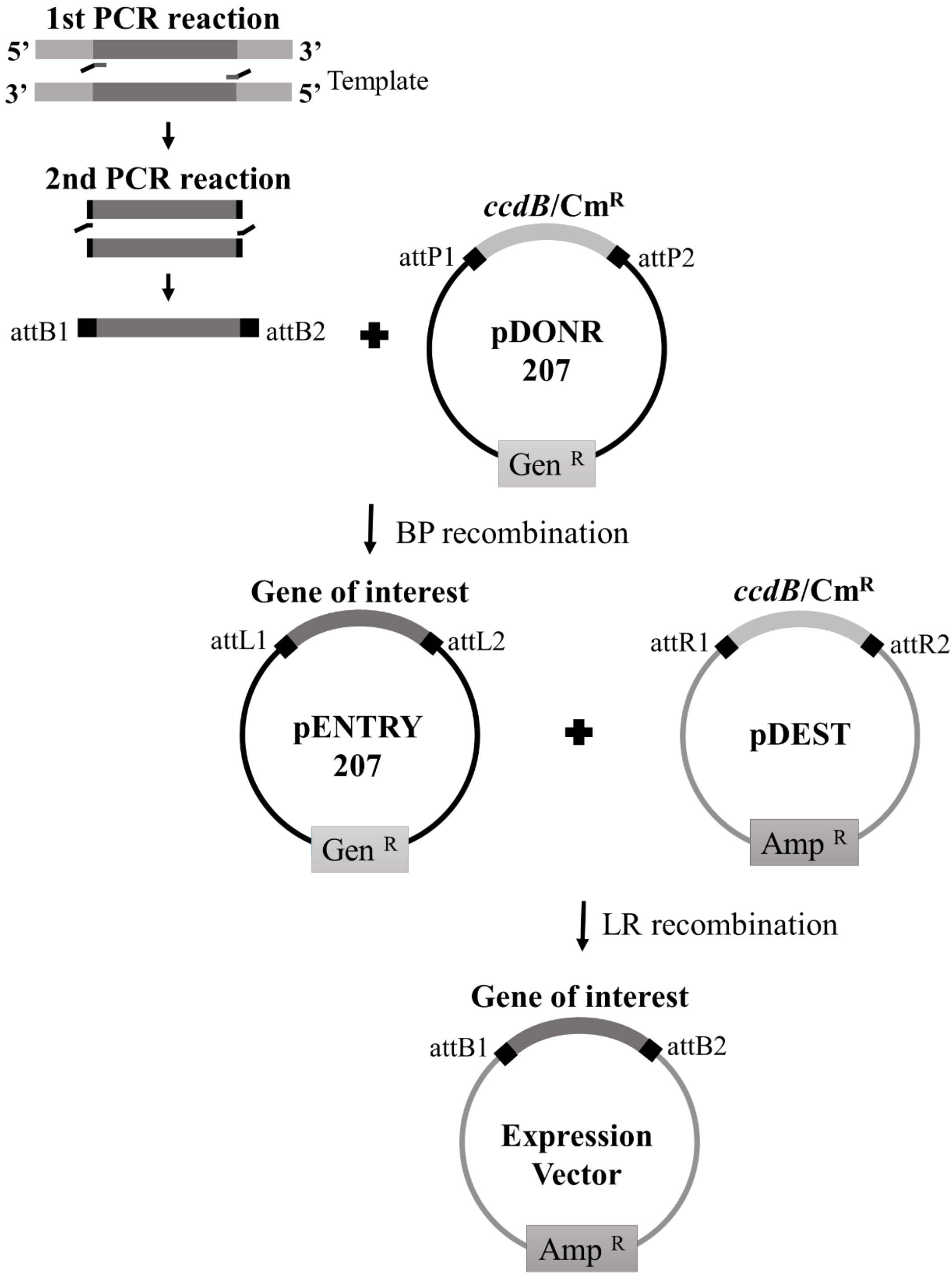

Figure S1. Gateway Cloning. Nested PCR and site-specific recombination reactions to obtain the bacterial expression vector with the gene of interest. Initially, we used primers consisting of a specific sequence to amplify the target sequence added with a sequence corresponding to half of the att1/att2 sites. Then, a nested PCR was done to complete the att sites. The BP recombination allows to insert a gene of interest into a so-called DONR vector to construct a library compatible with Gateway Cloning and several Destination vectors. In a second reaction called LR, the expression vector contains the gene of interest and is ready to use. All steps were controled by restriction analysis. pDONR vector inserts

were controled by sequencing. The double selection using antibiotics and *ccdB* sensible strains (*E. coli* DH5α or DH10B) is responsible for the high efficience of this technology.

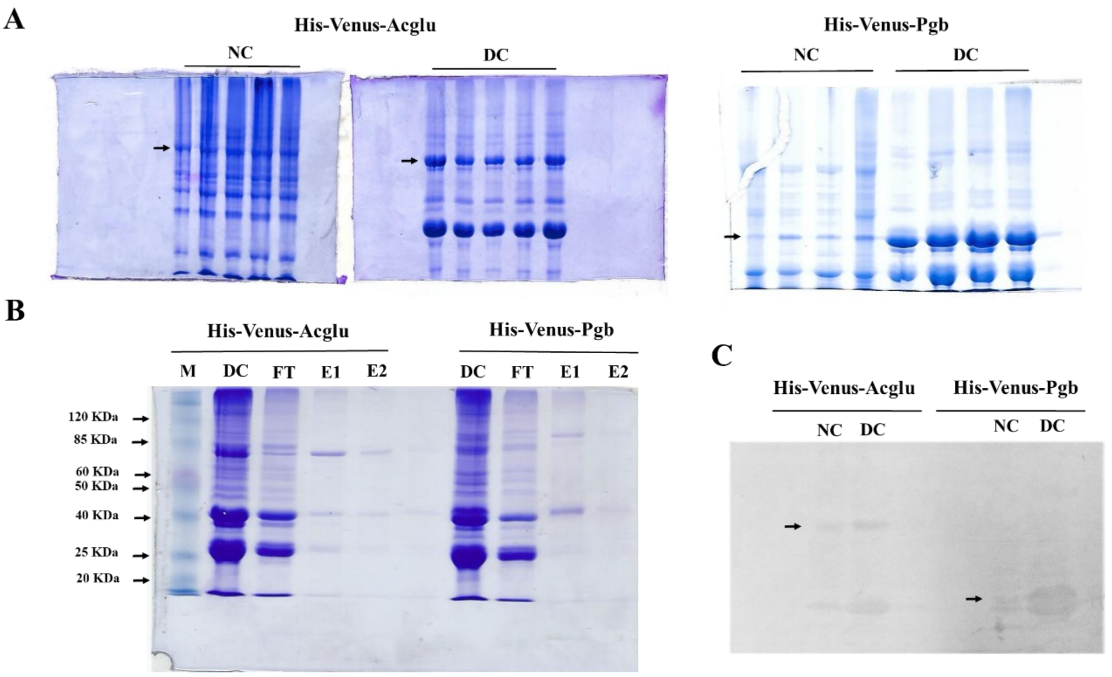

Figure S2. Expression of recombinant His-Venus-LysM proteins. (A) SDS-PAGE of His-Venus-Acglu and His-Venus-Pgb expressed in *E. coli* Rosetta after induction with 2 mM IPTG under native (NC) or denaturing (DC) conditions. **(B)** SDS-PAGE of His-Venus-Acglu and His-Venus-Pgb proteins purified under denaturing conditions using NiNTA chromatography. M: Pre-stained protein ladder; FT: Flow through; E1: first eluate; E2: second eluate. **(C)** Western blotting of the His-Venus-Acglu and His-Venus-Pgb recombinant proteins using a primary anti-RGS-His antibody. NC: native conditions. DC: denaturing conditions.

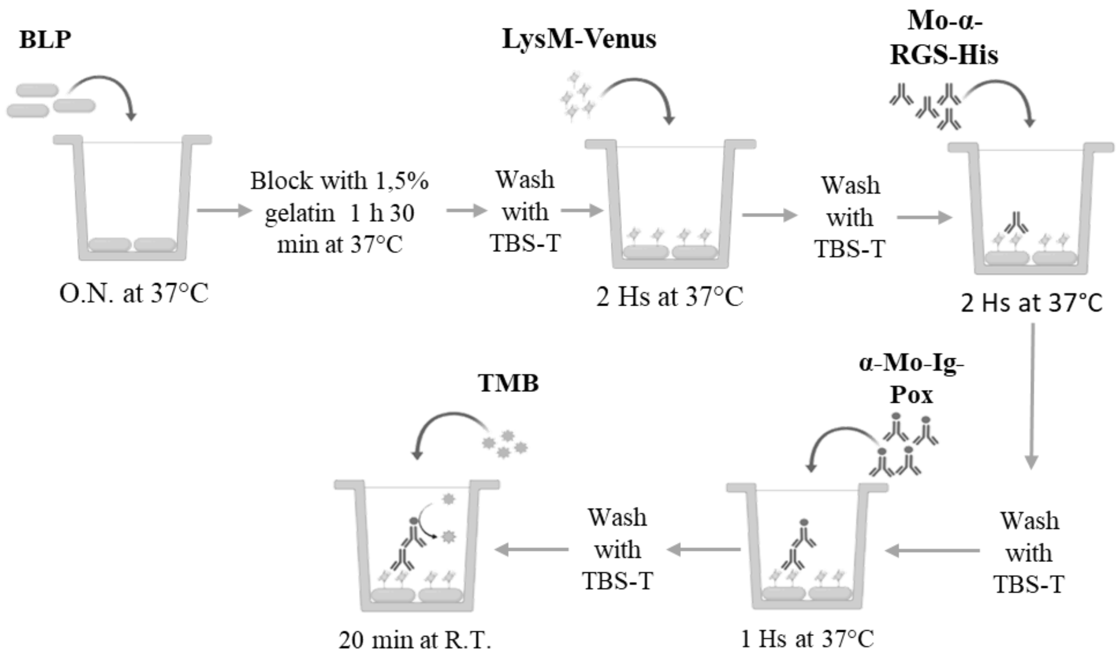

Figure S3. ELISA-based technique to detect the binding affinity of Venus-Acglu and Venus-Pgb proteins to BLPs from *Lacticaseibacillus rhamnosus* IBL027 (BLPs027).

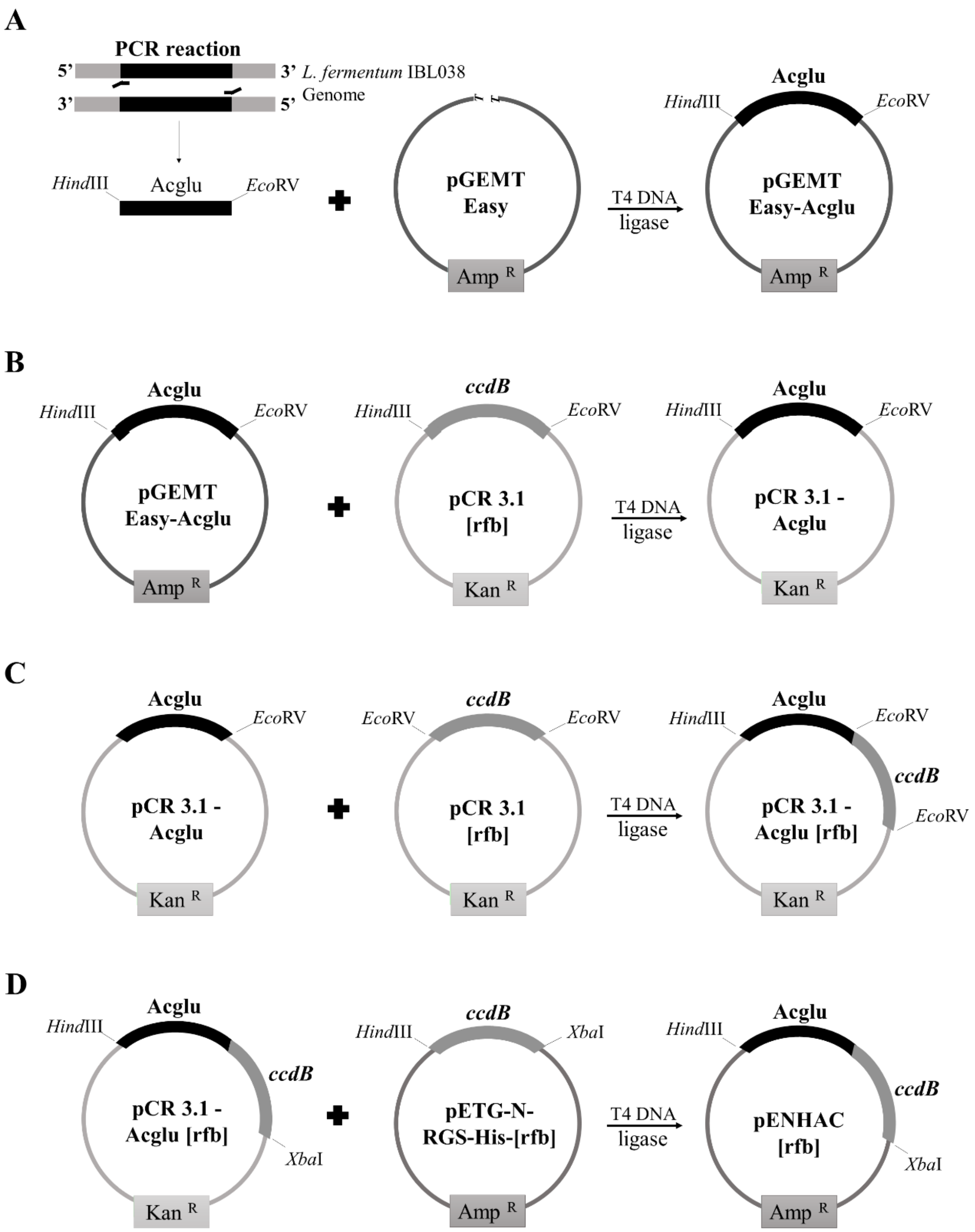

Figure S4. Schematic representation of the cloning strategy followed to construct a destination vector with the custom cassette 5'-*Hind*III-ATG-[RGS-His-tag-Acglu]-*Eco*RV-[ccdB/CmR (rfB)]-*Eco*RV-*Xba*I- 3 'compatible with the Gateway cloning technology. (A) PCR to amplify the domains encoding the five LysM domains in Acglu using *L. fermentum* IBL038 genomic DNA as a template. The purified PCR product and the plasmid pGEMT-Easy (Promega) were ligated using the enzyme T4 DNA ligase (New England Biolabs) according to the manufacturer's instructions. **(B)** First, the plasmids were digested enzymatically using the restriction enzymes shown in the figure. Then, a ligation reaction of the fragments corresponding to Acglu and the backbone of the plasmid pCR 3.1, obtained by enzymatic restriction and purified on agarose gels using a commercial kit (ADN Puriprep GP-Kit, InbioHighway, Argentina), using the enzyme T4 DNA ligase (New England Biolabs) **(C)** Similar to the step before, the plasmids were digested with *Hind*III/*Xba*I, purified and ligated. The insertion of the fragment

corresponding to the Gateway cassette [*ccdB*/CmR (rfB)] in the plasmid pCR 3.1 Acglu was done by a ligation reaction with the enzyme T4 DNA ligase (New England Biolabs). **(D)** Again, in the last step, the plasmids were digested enzymatically, and gel purified to obtain backbone and insert. Insertion of the customized cassette 5 '-*Hind*III-ATG-[RGS-His-tag-Acglu]-*Eco*RV-[ccdB / CmR (rfB)]-*Eco*RV-*Xba*I-3', in the vector pETG-N-RGS-His-[rfb] was done using a T4 DNA ligase (New England Biolabs).

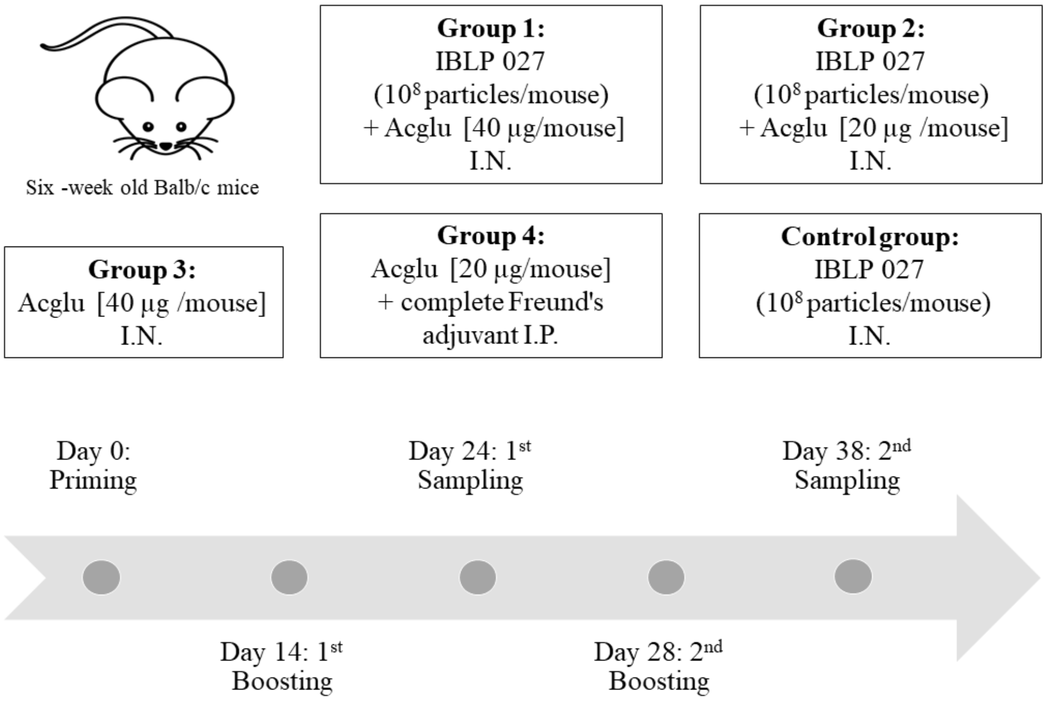

Figure S5. Immunization scheme. Mice divided in five groups for immunizations. Six-week old Balb/c mice were nasally immunized with Venus-Acglu$_{20}$-BLPs027 or Venus-Acglu$_{40}$-BLPs027 on days 0, 14(priming) and 28 (boosting). Mice nasally vaccinated (I.N.) with Venus-Acglu-His alone or intraperitoneally vaccinated (I.P.) with of Venus-Acglu-His with complete Freund's adjuvant were used for comparisons. Ten days after each boosting, serum, BAL and spleen samples were taken for analysis.

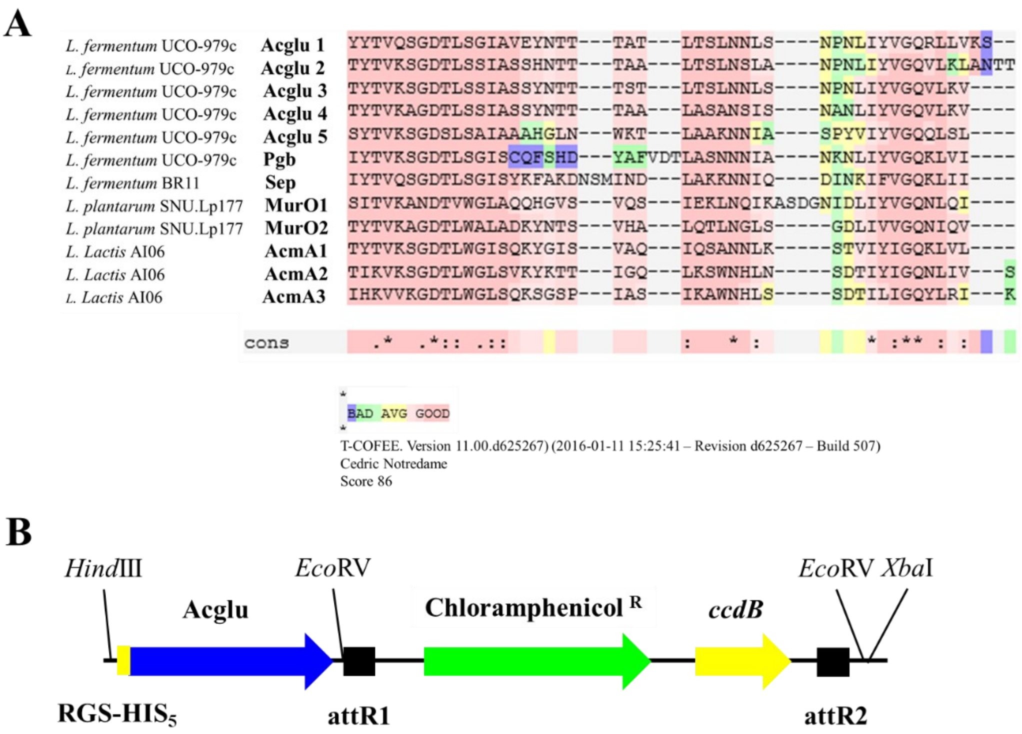

Figure S6. (A) Alignment of amino acid sequences among LysM motifs. Alignment of the three LysM motifs of AcmA (*Lactococcus lactis* AI06), the LysM motif of MurO (*Lactiplantibacillus plantarum* SNU.Lp177), the LysM motif of Sep *(Limosilactobacillus fermentum* BR11), the LysM motif of Pgb (*Limosilactobacillus fermentum* UCO-979C) and all five LysM motifs of Acglu (*L. fermentum* UCO-979C) performed with the program T-COFFEE (version 11.00.d625267). The final alignment is colored in function of the CORE index from pink (very consistent) to blue (poorly consistent). The consensus sequence is indicated below the alignment: * indicates positions which have a single and fully conserved residue, : indicates conservation between groups of strongly similar properties (scoring > 0.5 in the Gonnet PAM 250 matrix), and . indicates conservation between groups of weakly similar properties (scoring ≤ 0.5 in the Gonnet PAM 250 matrix). **(B) Scheme of pENHAC-[rfB] expression vector.** This vector is compatible with the Gateway cloning technology, in which the Acglu sequence was added to the N-terminal of the *ccdB*. This Figure does not claim proportional correctness.
